# The effect of parental psychological flexibility on children’s behavioral problems: a moderated mediation model

**DOI:** 10.1186/s13034-025-00863-y

**Published:** 2025-02-13

**Authors:** Jia Wang, Ru-De Liu, Jingmin Lin

**Affiliations:** 1 Teachers’ College, Beijing Union University, Beijing, People’s Republic of China; 2 Beijing Key Laboratory of Applied Experimental Psychology, National Demonstration Center for Experimental Psychology Education, Faculty of Psychology, Beijing Normal University, Beijing, People’s Republic of China; 3 Beijing Key Laboratory of Applied Experimental Psychology, Faculty of Psychology, Beijing Normal University, Beijing, People’s Republic of China

**Keywords:** Parental psychological flexibility, Parent-child conflict, Parental phubbing, Internalizing problems, Externalizing problems

## Abstract

**Background:**

Building upon previous research suggesting that parental psychological flexibility is negatively associate with children’s behavioral problems, this study examined a moderated mediation model to explore the effect of parental psychological flexibility on children’s internalizing and externalizing problems. Specifically, parent-child conflict is considered as a mediator while parental phubbing is examined as a moderator.

**Methods:**

This study employed a cross-sectional design, with a total of 1060 parents of preschool-aged children participating. The parents completed a series of surveys, including the Parental Psychological Flexibility Questionnaire, the short form of the Child–Parent Relationship Scale, the Parental Phubbing Scale, and the Strengths and Difficulties Questionnaire regarding their children. The moderated mediation model was assessed using SPSS PROCESS 4.1.

**Results:**

The results indicated that parental psychological flexibility negatively predicted children’s behavioral problems through the mediating effect of parent-child conflict, with this relationship being moderated by parental phubbing; an increase in phubbing weakened the negative correlation between parental psychological flexibility and parent-child conflict, thereby further weakened the negative correlation between parental psychological flexibility and children’s behavioral problems.

**Conclusion:**

These findings offer potential strategies for parents to mitigate the risk of their children developing internalizing or externalizing problems, by enhancing parental psychological flexibility and reducing instances of phubbing behavior.

## Background

Internalizing and externalizing problems commonly begin during early childhood [1]. Recently, there has been a discernible upward trajectory in the occurrence of behavioral problems among children [2, 3]. Parental psychological flexibility (PPF), as a key indicator of parental mental health, has been proposed to negatively predict children’s behavioral problems. Specifically, children with parents exhibiting high levels of PPF are less susceptible to developing behavioral problems [4, 5]. The underlying mechanisms of the impact of PPF on children’s behavioral problems remain inadequately explored, encompassing both the precise mechanism through which PPF influences these problems and the specific conditions that optimize its effectiveness. The current study aimed to tackle these questions by exploring the mediating and moderating mechanism underlying the association between PPF with children’s internalizing and externalizing problems.

### The effect of PPF on children’s behavioral problems

Psychological flexibility is a highly predictive determinant of parental mental well-being [6]. Psychological flexibility in parenting situations (PPF) refers to the acceptance of negative thoughts, feelings, and impulses by parents during the process of child-rearing, while concurrently employing effective parenting strategies to foster a positive parent-child relationship [7]. PPF is an internal skill that enables parents to effectively navigate stressful events in the realm of parenting [8]. Lack of PPF is related to poor parental psychological adjustment [9, 10] and dysfunctional parenting strategies, such as over-reaction and permissive parenting style [4, 7]. In contrast, parents with higher levels of PPF may experience a reduction in parenting-related stress [8, 11], leading to improved mental well-being [12] and the adoption of an authoritative parenting style [13], consequently contributing to a decrease in children’s behavioral problems [14].

Empirical evidence from studies conducted with children and adolescents indicates that PPF demonstrates efficacy in mitigating internalizing problems, encompassing emotional difficulties, as well as externalizing problems, including aggressive behaviors and attention problems [5, 12]. For example, a cross-sectional study examined the association between PPF and internalizing and externalizing problems in children aged 3–18, revealing that higher levels of PPF were significantly linked to reduced occurrences of these problems [5]. In a longitudinal investigation, it was found that parental psychological inflexibility had a direct impact on their children’s internalizing and externalizing difficulties [12]. The findings of these empirical studies suggest that PPF may have a direct impact on children’s behavioral problems.

Besides the direct effect, PPF also influences children’s behavioral problems through the mediating role of other variables. The definition of PPF proposes that a high level of PPF signifies dual parental capacities: engaging in positive parenting practices and fostering a positive parent-child relationship [5, 7]. Both components within this definition—parenting practices and the parent-child relationship—have significant impacts on children’s behavioral problems [4, 15, 16]. Therefore, it is reasonable to infer that PPF may influence children’s behavioral problems through the mediating effects of parenting practices and the parent-child relationship. Conducting empirical research to investigate these two mediating effects would contribute to a more comprehensive understanding and support for the definition of PPF, as well as a deeper insight into the underlying mechanisms through which PPF influences children’s behavioral problems. Recently, empirical evidence has demonstrated the mediating effect of parenting practices between PPF and children’s behavioral problems [4]. However, there is still a dearth of empirical studies investigating the potential mediating role of the parent-child relationship in linking PPF to children’s behavioral problems. Therefore, this study will examine this mediating effect.

### The mediating effect of parent-child conflict

The parent-child relationship encompasses two components, namely parent-child conflict and parent-child intimacy, both of which demonstrate a strong and direct correlation with children’s behavioral problems [17, 18]. The relationship between these dimensions and children’s behavioral problems may vary across cultures, with parent-child conflict exhibiting a stronger correlation with children’s behavioral problems in collectivistic cultures [15]. Considering that the present study is conducted within a collectivist cultural framework, this study will solely focus on examining the impact of parent-child conflict on children’s behavioral problems.

Parent-child conflict is identified as a prominent risk factor contributing to the manifestation of behavioral problems in children [15, 16]. On one hand, parent-child conflict engenders a sense of diminished parental affection and support among children [19], thereby augmenting the susceptibility to internalizing problems including impaired peer engagement and emotional disturbances [20–22]. On the other hand, conflicts with parents may lead to alienation and defiance in children [18], thereby amplifying the risks of externalizing problems such as irritability and aggression [23, 24].

As posited by the theoretical framework of Acceptance and Commitment Therapy (ACT), PPF plays a pivotal role in shaping parenting behaviors and influencing parent-child interactions [25]. Receiving parent counseling in ACT can help parents enhance their PPF, thereby fostering an improved parent-child relationship [26]. Parents with low PPF may employ overprotective parenting behaviors to shield their children from harm [27], or adopt stricter discipline and exert greater control in order to prevent their children from making mistakes [4, 28]. However, these approaches may inadvertently elicit rebellious behaviors in the child, leading to heightened parent-child conflicts. Conversely, parents with high PPF tend to exhibit enhanced sensitivity towards their children’s needs and offer more suitable and adaptable support, thereby fostering higher-quality parent-child interactions [29]. Empirical studies have also provided evidence that PPF has a significant impact on parent-child conflict. A meta-analysis revealed a negative association between higher levels of parental flexibility and family conflict, while demonstrating greater family cohesion [8]. Additionally, Li et al. (2022) found in their cross-sectional study that PPF predicted children’s anxiety through the mediating effect of father-child attachment [29]. In summary, it is reasonable to infer that parent-child conflict may mediate the relationship between PPF and children’s internalizing and externalizing problems.

### The moderating effect of parental phubbing

PPF exhibits a negative correlation with children’s behavioral problems; however, this does not necessarily mean that higher levels of PPF in parents will result in a proportional reduction in children’s behavioral problems. Previous studies have suggested that other variables may influence how effectively PPF translates into adaptive parenting practices and child outcomes [30, 31]. A Chinese study found that the exposure to contradictions in the grandparent-parent co-parenting relationship moderates the influence of PPF on children’s emotion regulation [30]. Specifically, high exposure to contradictions reduces the positive effect of PPF on children’s emotional regulation skills compared to low exposure. Another study examined the relationship between PPF and parenting self-efficacy and found that work-family conflict moderates this relationship [31]. High levels of work-family conflict weaken the positive association between PPF and parenting efficacy. These studies demonstrated that extent of PPF’s impact on children’s outcomes varies under different conditions. Identifying the factors that moderate PPF’s effects during clinical interventions is crucial for optimizing outcomes.

In contemporary society, smartphones have become an indispensable tool in daily life, with parents commonly utilizing them while accompanying their children [32]. Consequently, parental phubbing has emerged as a new common negative parenting behavior and has been demonstrated its moderating effect on the relationship between other parenting behaviors and children’s outcomes [33, 34]. Specifically, high levels of parental phubbing weakened the positive impact of parental active mediation on children’s self-control attitude and smartphone dependency [34]. This study aims to offer a nuanced understanding of how digital-era parenting practices intersect with fundamental psychological constructs to influence children’s development, particularly by examining whether parental phubbing undermines the positive effects of PPF. Parental phubbing is a phenomenon where parents become absorbed in smartphones, leading to distractions during interactions with children and subsequently evoking feelings of neglect and exclusion among the latter [34, 35]. Phubbing behaviors may diminish parents’ responsiveness and concern towards their offspring [36, 37], thereby disrupting parent-child interactions and increasing parent-child conflict [38–40]. Moreover, empirical evidence has demonstrated that parental phubbing exerts significant effects on children’s mental health and is an important contributor to behavioral problems in children [40–42].

Parental phubbing may serve as a moderator in the relationship between PPF and parent-child conflict, as well as children’s behavioral problems, for several reasons. Firstly, although high PPF endows parents with the emotional and cognitive resources necessary to manage conflict and behavioral challenges, phubbing can create an interpersonal barrier that hinders the full utilization of these resources [43]. For example, even a psychologically flexible parent may fail to provide timely emotional support if their attention is frequently diverted to their smartphones. Consequently, phubbing behaviors may weaken the effect of PPF on reducing both parent-child conflict and children’s behavioral problems.

Secondly, based on the Interpersonal acceptance-rejection theory (IPARTheory), parental acceptance-rejection, as a primary form of parenting, has a significant impact on children’s psychological outcomes [44]. Previous research has indicated that within a family, parental acceptance (i.e. warmth, love, et al.) and parental rejection (i.e. neglect, abuse, et al.) can coexist and have an interactive impact on children’s behavioral problems. Specifically, receiving both acceptance and rejection from parents may engender confusion in children regarding parental love, and undermine their sense of security, thereby impeding the parent-child interaction and fostering behavioral problems [45–47]. As mentioned before, flexible parents are more likely to exhibit greater warmth and support (acceptance) towards their children [29], whereas parental phubbing is correlated with increased neglect and exclusionary behaviors (rejection) towards children [48]. Therefore, if parents with high PPF engage in phubbing behaviors, it can elicit a complex experience of both parental acceptance and rejection in their children, thereby diminishing the predictive power of PPF for reducing parent-child conflict and children’s behavioral problems.

Given the aforementioned reasons, parental phubbing may moderate the effect of PPF on parent-child conflict and children’s internalizing/externalizing problems. Specifically, when parents exhibit higher levels of phubbing behaviors, higher PPF may not predict less parent-child conflict and children’s behavioral problems compared to situations where parents display lower levels of phubbing behaviors.

### The present study

**Fig. 1:**
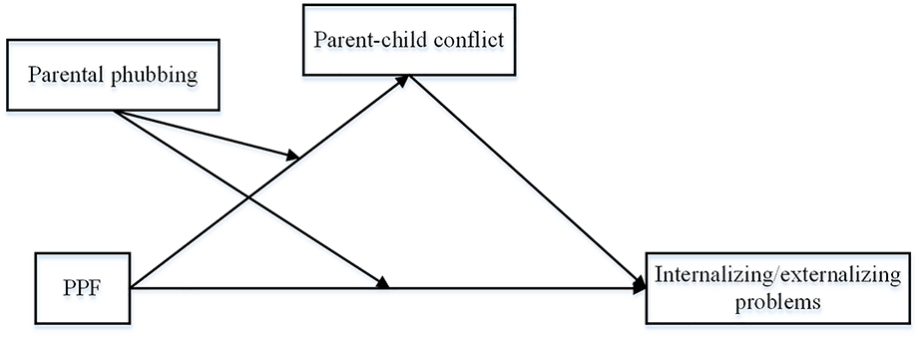
The hypothesized model

This study aimed to investigate the impact of PPF on children’s internalizing/externalizing problems, as well as its underlying mechanism. As depicted in Fig. 1, this study hypothesized that PPF negatively predicts children’s internalizing/externalizing problems (H1), parent-child conflict mediates the association between PPF and children’s internalizing/externalizing problems (H2), parental phubbing moderates the direct effect of PPF on children’s internalizing/externalizing problems (H3), and parental phubbing also moderates the indirect effect of PPF on children’s internalizing/externalizing problems via parent-child conflict (H4).

## Methods

### Participants and procedure

Participants recruited from six kindergartens in Northeast China, including three public and three private kindergartens. We distributed survey recruitment information to all parents whose children were attending in these kindergartens. The recruitment materials stated that participation in the survey would yield a feedback report on their parenting practices and parent-child relationships. A total of 1060 parents voluntarily took part in this survey. Among the participants, 898 were mothers aged between 24 and 46 (*M* = 35.76, *SD* = 3.86), while the remaining 162 were fathers (15.28%) aged between 25 and 49 (*M* = 37.22, *SD* = 4.60). In terms of their children’s gender distribution, girls accounted for 47.3% (*N* = 501) and boys accounted for 52.7% (*N* = 559). The mean age of their children was 5.32 years old (*SD* = 1.04), ranging from 3 to 7 years. This study obtained approval from the Research Ethics Committee of a university in Beijing. Following the consent of both teachers and parents, the survey was carried out under the organization of the teachers. At the beginning of the survey, parents were requested to provide an email address for receiving the feedback report. Subsequently, they were asked to complete a series of questionnaires assessing PPF, parental phubbing, parent-child conflict, and their children’s behavioral problems. A total of 1060 valid questionnaires were amassed with a retrieval rate of 100%.

### Measures

*PPF.* The Chinese version of the 19-item Parental Psychological Flexibility Questionnaire (PPFQ) [7] was used to measure PPF. The PPFQ has been employed in different studies investigating PPF among parents with children aged 3–6 [11, 49, 50]. The questionnaire comprises three dimensions, namely cognitive defusion (8 items), committed action (5 items), and acceptance (6 items). In this study, two items on the original scale that are not suitable for preschool-aged children have been modified. Specifically, the statement “watching my child deal with new experiences as he/she grows up (e.g., starting high school, first kiss, puberty) is interesting and exciting” has been revised to “watching my child deal with new experiences as he/she grows up (e.g., commencing kindergarten, establishing initial friendships) is interesting and exciting.” Additionally, the statement “I have refused to let my child do things that were important to them because I would worry too much (e.g., spend time with friends, walk to school by themselves)” has been modified to “I have refused to let my child do things that were important to them because I would worry too much (e.g., spend time with friends).” We replaced the examples in these original items with ones that are more appropriate for preschoolers while maintaining the wording and meaning of the items. The measurement of all items was conducted using a 7-point Likert scale (1 = *never true* to 7 = *always true*), with the cognitive defusion and committed action dimensions featuring reversed items. Higher scores indicated elevated levels of PPF among parents. The CFA results indicated good validity of the scale employed in this study (*χ*^*2*^*/df* = 3.22, CFI = 0.984, TLI = 0.978, RMSEA = 0.046, SRMR = 0.049). The Cronbach’s *α* coefficient for the overall scale was found to be 0.90, while ranging from 0.72 to 0.95 for the subscales.

*Parent-child conflict.* Parent-child conflict was measured using the conflict subscale of the short form of Child–Parent Relationship Scale (CPRS-SF) developed by Driscoll and Pianta [51]. The subscale comprised 8 items, each of which was assessed using a 5-point Likert scale (1 = *definitely does not apply* to 5 = *definitely applies*). Higher scores indicated a greater degree of conflict between parents and children. The Cronbach’s *α* of the conflict subscales in this study was 0.83.

*Parental phubbing.* The measure of parental phubbing in this study was derived from the 7-item Perceived Parental Phubbing Scale (PPS) [52]. Based on previous study [53], the references to “my mother/my father” in the original scale were substituted with “I” in this study, to transform the scale into a self-reported measure of parental phubbing behavior. For example, “My mother/father get distracted by his/her smartphone when we do something together” was changed into “I get distracted by my smartphone when my child and I do something together”. The parents were asked to rate each item on a 5-point Likert scale ranging from 1 (*never*) to 5 (*all the time*), with higher scores indicating elevated levels of phubbing behavior. The Cronbach’s *α* in this study was 0.85.

*Children’s behavioral problems.* Parents completed the Strengths and Difficulties Questionnaire (SDQ) [54] to assess their children’s behavioral problems. Behavioral problems in the SDQ were assessed across four dimensions, namely emotional symptoms, peer relationship problems, conduct problems, and hyperactivity. Each dimension comprised five items that were rated on a 3-point Likert scale (0 = *not true*, 1 = *somewhat true*, 2 = *certainly true*). Following the approach suggested by Goodman et al. (2010), internalizing problems were calculated as the sum of the ten items from the first two dimensions (emotional symptoms & peer relationship problems), while externalizing problems were determined by summing the ten items from the remaining two dimensions (conduct problems & hyperactivity) [55]. Higher scores were indicative of a greater presence of behavioral problems. The Cronbach’s *α* coefficients for the overall scale, internalizing subscale, and externalizing subscale in this study were 0.78, 0.65, and 0.74 respectively.

### Data analysis

The descriptive statistics, correlation analyses, and Harman’s one-factor test were conducted using SPSS 20. Subsequently, the mediation effect of parent-child conflict on the relationship between PPF and internalizing/externalizing problems was examined using SPSS PROCESS 4.1 (Model 4). Finally, the moderated mediation model was assessed using SPSS PROCESS 4.1 (Model 8). The confidence intervals (CI) were estimated using bootstrapping with a sample size of 5000.

## Results

### Common method bias test

The results of Harman’s one-factor test revealed that ten common factors had initial eigenvalues exceeding 1, with the first common factor accounting for 21.68% (< 40%) of the variance. Consequently, potential concerns regarding common method bias were deemed negligible in this study.

### Preliminary analyses

The results of descriptive and correlation analyses are reported in Table 1. PPF was negatively related to parent-child conflict, parental phubbing, children’s internalizing and externalizing problems. Parent-child conflict was positively correlated with children’s internalizing problems and externalizing problems. Additionally, parental phubbing was positively associated with parent-child conflict, children’s internalizing and externalizing problems.

**Table 1:** Descriptive statistics and correlations for main variables

Variable	Statistic		Correlations			
	M	SD	1	2	3	4
1. PPF	5.35	1.01	–			
2. Parent-child conflict	1.97	0.73	– 0.58**	–		
3. Parental phubbing	2.48	0.75	– 0.23**	0.18**	–	
4. Internalizing problem	4.05	2.70	– 0.33**	0.41**	0.15**	–
5. Externalizing problem	6.16	3.14	– 0.28**	0.46**	0.20**	0.42**

*p < 0.05, **p < 0.01

### Mediation analyses

The mediation analysis results are shown in Table 2. After controlling for the gender and age of children, as well as parental identity (father vs. mother), PPF was found to have a statistically significant negative association with internalizing problems (direct effect =– 0.14, 95% CI [– 0.21,– 0.07]). Furthermore, this relationship was mediated by parent-child conflict (indirect effect =– 0.19, 95% CI [– 0.21,– 0.07]), accounting for 56.85% of the total effect of PPF on internalizing problems. Regarding children’s externalizing problems, the direct effect of PPF was not significant (direct effect =– 0.03, 95% CI [– 0.09, 0.04]). However, there was a significant indirect effect through parent-child conflict (indirect effect =– 0.26, 95% CI [– 0.30,– 0.21]), which accounted for 90.90% of the total effect. These results provide partial support for hypothesis H1 and complete support for hypothesis H2.

**Table 2:** Results of the mediation models

Outcomes	Predictors	β	SE	t	95% CI
IP	Gender	0.01	0.06	0.23	[– 0.10, 0.12]
	Age	– 0.07*	0.03	– 2.56	[– 0.13,– 0.02]
	PI	– 0.17*	0.08	– 2.17	[– 0.31,– 0.02]
	PPF	– 0.14***	0.03	– 4.15	[– 0.21,– 0.07]
	PCC	0.32***	0.03	9.51	[0.26, 0.39]
	*R* ^*2*^	0.19***			
	*F*	49.24			
EP	Gender	– 0.16**	0.05	– 2.87	[– 0.26,– 0.05]
	Age	– 0.05^†^	0.03	– 1.78	[– 0.10, 0.01]
	PI	– 0.07	0.08	– 0.87	[– 0.21, 0.08]
	PPF	– 0.03	0.03	– 0.77	[– 0.09, 0.04]
	PCC	0.45***	0.03	13.40	[0.38, 0.51]
	*R* ^*2*^	0.22***			
	*F*	60.92			

*PI* parental identity, *PPF* parental psychology flexibility, *PCC* parent-child conflict, *IP* internalizing problem, *EP* externalizing problem

*p < 0.05, **p < 0.01, ***p < 0.001

^†^*p* < 0.10

### Moderated mediation analyses

**Fig. 2:**
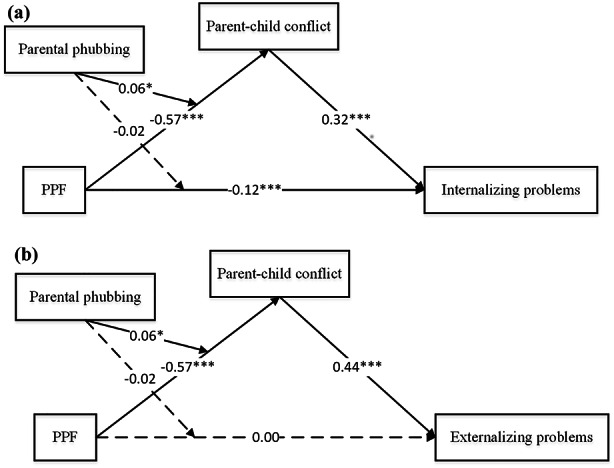
**a** Path coefficients of the model with internalizing problem as the outcome. *p < 0.05, **p < 0.01, ***p < 0.001. **b** Path coefficients of the model with externalizing problem as the outcome. *p < 0.05, **p < 0.01, ***p < 0.001

**Fig. 3:**
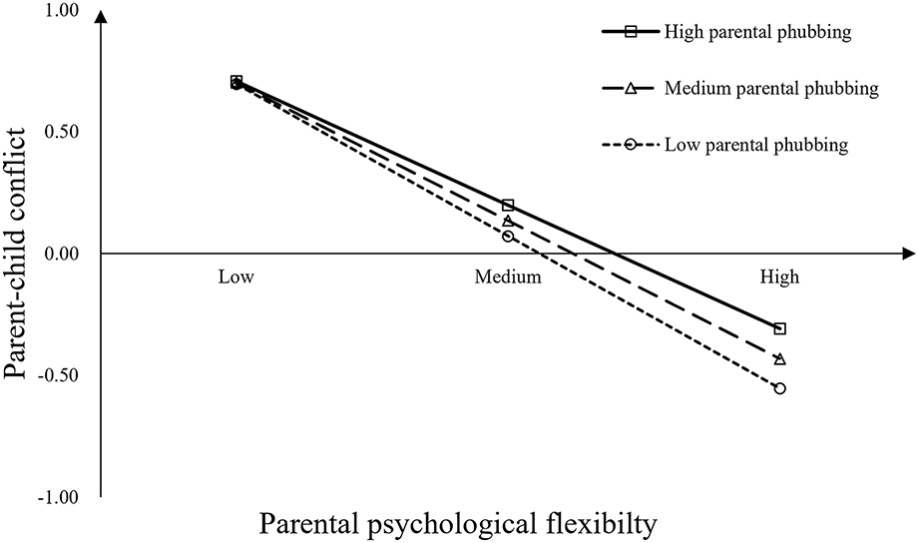
The moderating effect of parental phubbing on the association between PPF and parent-child conflict

The results of the moderated mediation analyses are presented in Table 3, while Fig. 2 illustrates the coefficients of the hypothesized paths. After controlling for children’s gender, age, and parental identity (father vs. mother), the moderating effect of parental phubbing on the direct pathway between PPF and internalizing problems (*β* =– 0.02, *p* = 0.485), as well as between PPF and externalizing problems (*β* =– 0.02, *p* = 0.442) were nonsignificant. Therefore, H3 was not supported. The relationship between PPF and parent-child conflict was found to be moderated by parental phubbing (*β* = 0.06, *p* < 0.05). As depicted in Fig. 3, when parental phubbing was high (+ 1 SD), the effect of PPF on parent-child conflict weakened (*β* =– 0.51, *p* < 0.001) compared to when it was low (– 1 SD; *β* =– 0.63, *p* < 0.001). Furthermore, conditional indirect effect analysis revealed that parental phubbing moderated the mediation effect of parent-child conflict on the association between PPF and internalizing problems (Index = 0.02, *SE* = 0.01, 95% CI [0.001, 0.04]), as well as on the association between PPF and externalizing problems (Index = 0.03, *SE* = 0.01, 95% CI [0.002, 0.05]). H4 received support.

**Table 3:** Results of the moderated mediation models

	Outcomes	Oredictors	β	SE	t	95% CI
Model 1	PCC	Gender	0.01	0.05	0.19	[– 0.09, 0.11]
		Age	– 0.03	0.03	– 1.17	[– 0.08, 0.02]
		PI	– 0.15*	0.07	– 2.14	[– 0.29,– 0.01]
		PPF	– 0.57***	0.03	– 21.92	[– 0.62,– 0.52]
		PP	0.06*	0.03	2.46	[0.01, 0.12]
		PPF × PP	0.06*	0.02	2.43	[0.01, 0.11]
		*R* ^*2*^	0.34***			
		*F*	90.55			
	IP	Gender	0.01	0.06	0.21	[– 0.10, 0.12]
		Age	– 0.07**	0.03	– 2.68	[– 0.13,– 0.02]
		PI	– 0.17*	0.08	– 2.22	[– 0.32,– 0.02]
		PPF	– 0.12***	0.03	– 3.62	[– 0.19,– 0.06]
		PP	0.07*	0.03	2.37	[0.01, 0.13]
		PCC	0.32***	0.03	9.38	[0.25, 0.39]
		PPF × PP	– 0.02	0.03	– 0.7	[– 0.07, 0.03]
		*R* ^*2*^	0.19***			
		*F*	36.29			
Model 2	PCC	Gender	0.01	0.05	0.19	[– 0.09, 0.11]
		Age	– 0.03	0.03	– 1.17	[– 0.08, 0.02]
		PI	– 0.15*	0.07	– 2.14	[– 0.29,– 0.01]
		PPF	– 0.57***	0.03	– 21.92	[– 0.62,– 0.52]
		PP	0.06*	0.03	2.46	[0.01, 0.12]
		PPF × PP	0.06*	0.02	2.43	[0.01, 0.11]
		*R* ^*2*^	0.34***			
		*F*	90.55			
	EP	Gender	– 0.16**	0.05	– 2.96	[– 0.27,– 0.05]
		Age	– 0.05*	0.03	– 1.99	[– 0.11,– 0.00]
		PI	– 0.07	0.08	– 0.96	[– 0.22, 0.08]
		PPF	0.00	0.03	– 0.02	[– 0.07, 0.07]
		PP	0.12***	0.03	4.13	[0.06, 0.17]
		PCC	0.44***	0.03	13.22	[0.37, 0.50]
		PPF × PP	– 0.02	0.03	– 0.77	[– 0.07, 0.03]
		*R* ^*2*^	0.24***			
		*F*	46.91			
Conditional indirect effect: PPF→PPC→IP						
				*β*	*SE*	95% CI
		M– 1 SD		– 0.20	0.03	[– 0.25,– 0.15]
		M		– 0.18	0.02	[– 0.22,– 0.14]
		M + 1 SD		– 0.16	0.02	[– 0.20,– 0.12]
Conditional indirect effect: PPF→PPC→EP						
				*β*	*SE*	95% CI
		M– 1 SD		– 0.27	0.03	[– 0.33,– 0.22]
		M		– 0.25	0.02	[– 0.29,– 0.21]
		M + 1 SD		– 0.22	0.02	[– 0.27,– 0.18]

*PI* parental identity, *PPF* parental psychology flexibility, *PCC* parent-child conflict, *PP* parental phubbing, *IP* internalizing problem, *EP* externalizing problem

*p < 0.05, **p < 0.01, ***p < 0.001

^†^*p* < 0.10

## Discussion

The present study examined the specific mechanisms underlying the association between PPF and children’s behavioral problems. The findings revealed that PPF can mitigate the risks of behavioral problems by reducing parent-child conflict. Furthermore, this effect is moderated by parental phubbing behavior. These results suggested the presence of a moderated mediation mechanism in the link between PPF and children’s behavioral problems.

Firstly, this study revealed a negative correlation between PPF and children’s internalizing/externalizing problems. These findings are consistent with previous research positing that higher PPF is linked to reduced emotional and behavioral difficulties in children [4, 5]. Parents who exhibit susceptibility to negative emotions and thoughts regarding parenting may exert a detrimental influence on their offspring [7]. However, if parents demonstrate acceptance of negative emotions and possess the ability to implement optimal parenting strategies, it is unsurprising that their children will experience enhanced mental well-being [4, 5].

Secondly, the present study investigated the mediating effect of parent-child conflict in the association between PPF and children’s behavioral problems. The findings showed a negative relationship between PPF and parent-child conflict, indicating that a high level of psychological flexibility in parenting is associated with reduced occurrences of parent-child conflict. This result aligns with a previous investigation conducted among adolescents, which revealed that PPF exhibits the potential to augment father-adolescent attachment [29]. Moreover, this outcome offers further empirical validation for the theoretical framework of Acceptance and Commitment Therapy, which posits that PPF plays a crucial role in enhancing the quality of parent-child interaction [25, 26]. The mediation analyses also revealed a positive association between parent-child conflict and internalizing/externalizing problems. Increased levels of parent-child conflict were found to elevate the risk of behavioral problems in children, aligning with previous studies that have focused on the influence of parent-child interaction on children’s behavioral problems [18, 19].

Furthermore, the results of the mediation analyses indicated that parent-child conflict served as a mediator in explaining the impact of PPF on children’s behavioral problems (H2 supported). The findings further substantiate the notion that parent-child interaction is pivotal to children’s psychological development and serves as a crucial mediator between parental characteristics and children’s outcomes [56, 57]. A similar result was found in research examining the association between PPF, attachment, and anxiety among children [29]. Although parent-child conflict mediated both the association between PPF and internalizing problems, as well as the association between PPF and externalizing problems, the underlying mechanisms appear distinct. Specifically, the direct effects of PPF on internalizing problems was significant, suggesting that parental psychological flexibility plays a key role in alleviating children’s emotional distress, such as anxiety, depression, and withdrawal. High PPF enables parents to respond to their child’s emotions and needs in an adaptive and accepting manner [58], which may create a supportive and validating emotional environment that helps children regulate their emotions and reduce the likelihood of internalizing problems. In contrast, the direct effect of PPF on externalizing problems was not significant, indicating that externalizing problems (such as defiant and disruptive behaviors) may be more strongly associated with parent-child conflict as a mediating factor. This aligns with previous research involving 615 parents, which showed that PPF influenced children’s behavioral problems indirectly through mediators, rather than through a direct pathway [4]. Furthermore, given evidence that internalizing problems can accumulate and lead to externalizing behaviors over time [59, 60], it is plausible that PPF indirectly contributes to externalizing problems through its impact on internalizing problems. This may explain why the direct effect of PPF on internalizing problems was significant, whereas its direct effect on externalizing problems was not observed.

Thirdly, the present study examined the moderating role of parental phubbing in order to gain a deeper understanding of when PPF may diminish children’s behavioral problems. The results indicated that parental phubbing significantly moderated the mediating effect of parent-child conflict on the relationships between PPF with internalizing problems and between PPF with externalizing problems (H4 supported). High levels of parental phubbing weakened the effect of PPF on children’s behavioral problems though the mediating effect of parent-child conflict. Even though parents can flexibly cope with parenting issues, their ability to effectively mitigate parent-child conflict and subsequently alleviate their children’s behavioral problems will be compromised when they are addicted to mobile phones and neglect their children.

However, the moderating effects of parental phubbing on the direct pathway from PPF on children’s internalizing and externalizing problems were both found to be insignificant (H3 was not supported). The results indicated that parental phubbing moderated the relationship between PPF and parent-child relational dynamics, rather than between PPF and children’s behavioral problems. PPF primarily reflects internal coping and emotional regulation abilities, which influence parenting practices and the quality of parent-child interactions [5, 7]. Phubbing, on the other hand, is a more specific external behavior that directly disrupts communication and the emotional connection between parents and children [61]. Consequently, phubbing is particularly significant in undermining the effect of PPF on reducing parent-child conflict, as it may signal parental disinterest or inattentiveness, leading to misunderstandings, frustration, or resentment from children. In contrast, the direct effect of PPF on children’s behavioral problems is likely mediated by broader and more stable parenting patterns, such as consistent discipline and problem-solving approaches [62]. The impact of these parenting dimensions on children’s behavioral problems may be less influenced by transient behaviors such as phubbing. Therefore, while parental phubbing moderates the relationship between PPF and parent-child conflict, it does not significantly alter the effect of PPF on children’s behavioral problems through other direct mechanisms.

This study provides implications for practical intervention for parents to better parenting their kids. Psychiatrists and mental health professionals can integrate our findings into clinical practice to tailor interventions that not only target the children’s behavioral problems but also address the family dynamics and parental behaviors that contribute to these issues. For instance, clinicians can develop and implement strategies based on Acceptance and Commitment Therapy to assist parents in improving their psychological flexibility, enabling them to better manage emotional reactions towards parenting challenges and respond more adaptively to their children’s needs. Additionally, our study emphasizes the significance of instructing parents about the potential negative impact of phubbing on their children’s behavior. Clinicians can provide guidance on establishing tech-free zones or designated times ensuring uninterrupted parent-child engagement without technological interference.

The following limitations should be noted. Firstly, as the main variables in this study were reported by parents, the findings just represent a preliminary exploration of their relationship. Particularly considering that phubbing is commonly perceived as a negative behavior, there may exist social desirability bias in parental self-reports. Future research could replicate these results by employing multi-informant reports (e.g., teachers’ observations or objective screen-time measures) to mitigate potential biases. Secondly, while the current study controlled for parental identity in the model, it is important to recognize that fathers and mothers might have differential effects on children’s behavioral problems [28]. Future research should endeavor to analyze the distinct impacts of fathers and mothers and elucidate the disparities between them. Additionally, other potential confounding factors, such as parental socioeconomic status, digital literacy, or work-related stress, could also affect parental behavior [5, 63, 64]. Consequently, future studies should further explore the influence of these variables. At last, the current study, as a cross-sectional research design, is limited in its ability to establish a causal relationship between variables. Further longitudinal research is necessary to examine the effects of previous PPF, parent-child conflict, and parental phubbing on subsequent children’s internalizing/externalizing problems using cross-lagged models, thereby establishing a causal relationship between these factors and children’s behavior problems.

## Conclusion

This study illuminates the underlying mechanism of the relationship between PPF and children’s behavioral problems. Firstly, the present study provides empirical evidence supporting the negative predictive role of PPF on children’s behavioral problems, mediated by parent-child conflict. Moreover, the current study first identifies parental phubbing as a moderator that influences the effect of PPF. The practical implications also deserve attention. This study offers parental guidelines on mitigating behavioral problems in children, highlighting the efficacy of enhancing psychological flexibility in parenting practices. Moreover, it is crucial for parents to recognize that possessing psychological flexibility alone is insufficient; they need to put down smartphones and prioritize quality time with their children.

## Data Availability

Data is available from the corresponding author upon reasonable request.
